# Botanical Items

**Published:** 1886-02

**Authors:** 


### BOTANICAL ITEMS.

A botanical crank classifies some well-known trees as follows:
The personal pronoun—Yew tree.
Always in debt—Willow tree.
Found at the soap factory—Tallow tree.
School girl's delight—Gum tree.
A good one to lean against—Staff tree.
A favorite with the dudes—Spruce tree.
The professional dead beat—Sponge tree.
The physican's appeal—Olive tree.
A thorough traitor—Judas tree.
An acquaintance of the ox team—Haw tree.
Found on the sea shore—Beech tree.
Inclined to go backwark—Crab tree.
Makes itself felt—Thorn tree.—*Ex.*

